# Insight into the mechanism of molecular recognition between human Integrin-Linked Kinase and Cpd22 and its implication at atomic level

**DOI:** 10.1007/s10822-022-00466-1

**Published:** 2022-07-23

**Authors:** Javier García-Marín, Diego Rodríguez-Puyol, Juan J. Vaquero

**Affiliations:** 1 Departamento de Química Orgánica y Química Inorgánica, Instituto de Investigación Química Andrés M. del Río (IQAR), Universidad de Alcalá (IRYCIS), Alcalá de Henares, 28805 Madrid, Spain; 2 Departamento de Química Biológica y Estructural, Centro de Investigaciones Biológicas, CIB-CSIC, C/Ramiro de Maeztu 9, 28040 Madrid, Spain; 3 Sección de Nefrología, Departamento de Medicina, Hospital Príncipe de Asturias, Universidad de Alcalá (IRYCIS), Alcalá de Henares, 28805 Madrid, Spain; 4 Fundación Renal Iñigo Álvarez de Toledo (FRIAT) y Instituto de Salud Carlos III (REDinREN), Madrid, Spain; 5 Present Address: Department of Chemistry, University of Bath, Claverton Down, Bath, BA2 7AX UK

**Keywords:** Integrin-linked kinase, ILK, Cpd22, Molecular dynamics, PELE, Pseudokinase

## Abstract

Pseudokinases have received increasing attention over the past decade because of their role in different physiological phenomena. Although pseudokinases lack several active-site residues, thereby hindering their catalytic activity, recent discoveries have shown that these proteins can play a role in intracellular signaling thanks to their non-catalytic functions. Integrin-linked kinase (ILK) was discovered more than two decades ago and was subsequently validated as a promising target for neoplastic diseases. Since then, only a few small-molecule inhibitors have been described, with the V-shaped pyrazole Cpd22 being the most interesting and characterized. However, little is known about its detailed mechanism of action at atomic level. In this study, using a combination of computational chemistry methods including PELE calculations, docking, molecular dynamics and experimental surface plasmon resonance, we were able to prove the direct binding of this molecule to ILK, thus providing the basis of its molecular recognition by the protein and the effect over its architecture. Our breakthroughs show that Cpd22 binding stabilizes the ILK domain by binding to the pseudo-active site in a similar way to the ATP, possibly modulating its scaffolding properties as pseudokinase. Moreover, our results explain the experimental observations obtained during Cpd22 development, thus paving the way to the development of new chemical probes and potential drugs.

**Graphical abstract:**

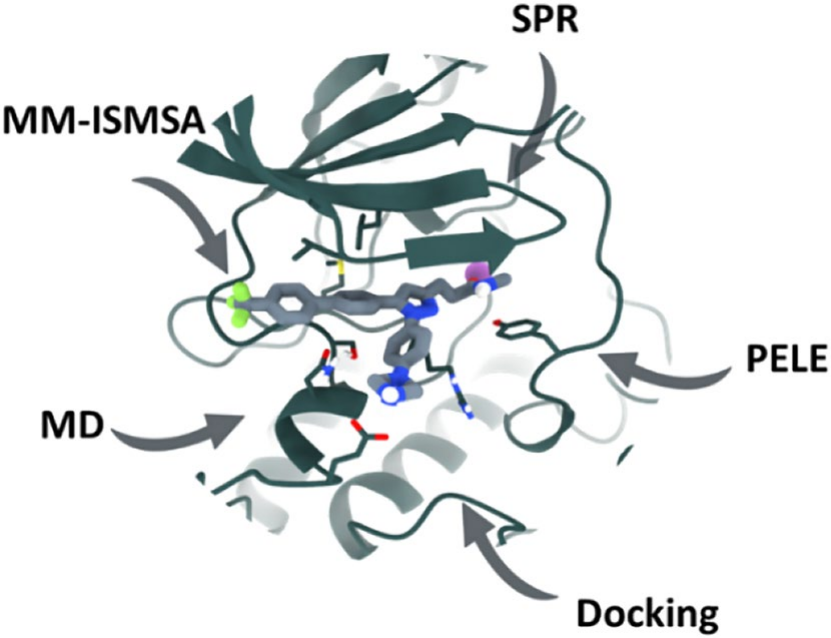

**Supplementary Information:**

The online version contains supplementary material available at 10.1007/s10822-022-00466-1.

## Introduction

Dynamic protein phosphorylation controlled by kinases and phosphatases is a key process in the regulation of cellular functions such as cell proliferation, apoptosis, and metabolism [1]. Protein kinases are quintessential signaling enzymes whose relation with different pathological imbalances has prompted numerous studies aimed at validating them as therapeutic targets for a variety of disorders, including cancer, inflammation, and viral diseases [1–4]. Since their discovery, pseudokinases have been considered to be evolutionary remnants of their parent proteins due to the absence of one or more residues at the pseudo-active site required for catalytic activity [5]. Notoriously, and in stark contrast to their counterparts, pseudokinases have received little attention as therapeutic targets. However, in the last decade, interest in them has increased due to the discovery of their role in cellular signaling via non-enzymatic functions such as scaffolding, allosteric modulation, and as protein-based switches [6–8].

Integrin-linked kinase (ILK), a protein that was discovered in 1996 by Hannigan and colleagues as an integrin β1 cytoplasmic tail-binding protein, is perhaps one of the most interesting members of the pseudokinase family [9]. ILK is a critical protein for survival and its constitutive loss results in lethality for mice [10]. Although ILK lacks the HRD and DFG motifs at its kinase-like *C*-terminal domain, there is a strong debate in the scientific community about its real behavior as a key serine-threonine kinase in integrin signaling [11–14]. ILK is a 51 kDa protein comprising 452 amino acids and three topological domains: the ankyrin repeat domain (residues 1 to 174), the pleckstrin-homology domain (175–192), and the kinase-like domain (amino acids 193 to 452) [15]. Thanks to these topological features, ILK interacts with parvin protein through its kinase-like domain and with the adaptor protein PINCH through the ankyrin repeats. In living organism, ILK is always associated to these two proteins constituting a tripartite complex known as IPP, a central component on the adesome network, which main function is linking integrins with actin cytoskeleton [16]. On the basis of the architecture of the kinase domain, ILK can be classified as a class 4 pseudokinase because of its ability to bind a nucleotide (not hydrolyzed ATP) and a cation (Mg^2+^ or Mn^2+^) [7]. This protein constitutes a signaling node inside the cell and, given its implication in multiple of physiological and pathological phenomena, it has been validated as a promising target for cancer [12, 17–21], as well as other diseases such as chronic kidney disease [22–24], inflammation [23, 25] or metabolic disorders [26].

**Fig. 1:**
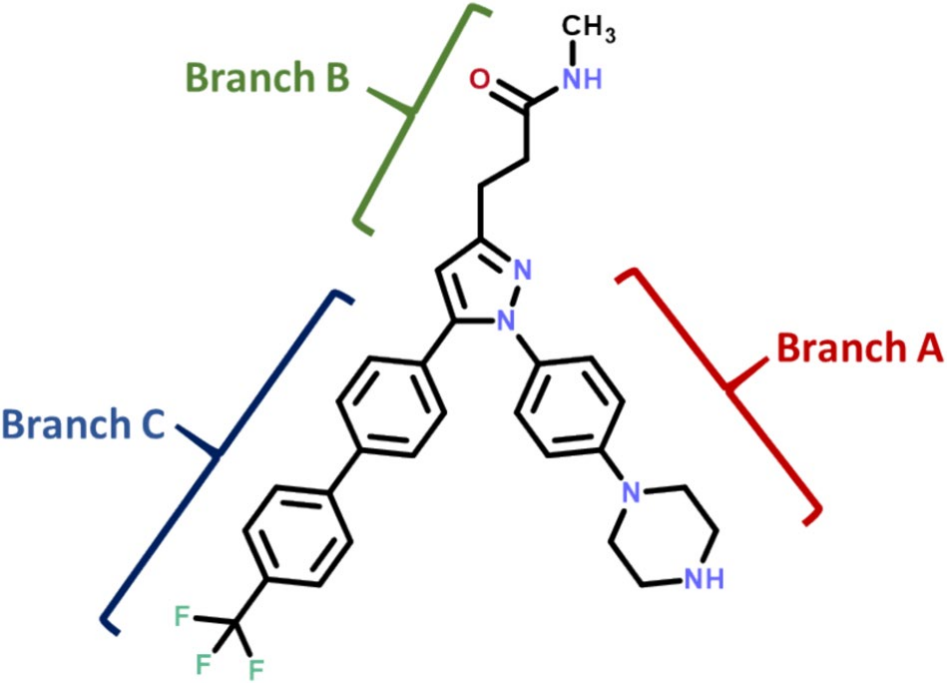
Chemical structure of ILK inhibitor Cpd22

The interest in developing ILK inhibitors has led to the discovery of a few promising drug-like candidates, including small-molecules and natural products [27, 28], with the V-shaped pyrazole Cpd22 being the most well-characterized and widely employed reference chemical probe against ILK given its commercial availability (Fig. 1) [29].

This molecule was discovered as result of a screening and lead-optimization campaign intended to identify potential kinase inhibitors. Cpd22 is a cell-permeable, drug-like compound that exhibit high in vitro potency against cell proliferation (IC_50_: 1–2.5 μM) and an antiproliferative effect by inducing both autophagy and apoptosis [29]. The mechanism of action of Cpd22 in vivo involves inhibition of the phosphorylation of two downstream ILK signaling proteins, namely GSK-3β and Akt, at residues Ser9 and Ser473, respectively [30]. Despite its interesting features, inhibition of ILK by Cpd22 has not been confirmed by in vitro biochemical or biophysical assays using recombinant ILK, supporting the molecular recognition phenomenon between both partners and their direct physical interaction. Moreover, to the best of our knowledge, structural data for the binding mode have not been published to date. A detailed understanding of the interaction of the ligand with its target at an atomic level is crucial for structure-based drug design as well as for structural chemical biology or biophysics and the impact of ligand binding on protein dynamics.

Given that Cpd22 has been shown to inhibit ILK in phenotypic assays, and the paramount need for a detailed understanding of the mechanism of action at the atomic level, we aimed to design a biomolecular modeling protocol to address these questions and to account for the experimental structure–activity relationships (SAR) reported in the literature.

## Results and discussion

### Assessment of the binding of Cpd22 to ILK by surface plasmon resonance

**Fig. 2:**
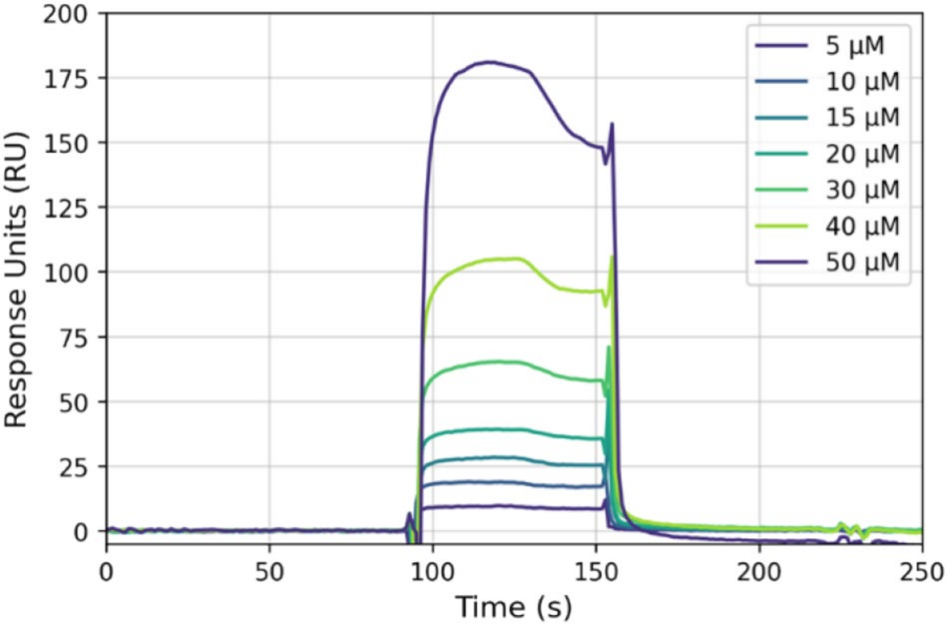
Sensogram for binding of compound Cpd22 to recombinant ILK using increasing concentrations of the analyte (Cpd22) in the range 5–50 μM (Chi^2^ = 1.65). The K_D_ was calculated by fitting the data to a single binding site model

Initially, to lay the groundwork for our subsequent simulations, we decided to test the hypothesis of direct ligand binding to ILK. In this regard, to gain both qualitative and quantitative insights, we carried out a surface plasmon resonance (SPR) assay to confirm Cpd22 ligation toward the protein (Fig. 2). Both Cpd22 and recombinant ILK were obtained from commercial sources. Using steady-state analysis of SPR measurements for the interaction between Cpd22 and ILK at room temperature (25 °C), the dissociation constant (K_D_) was adjusted by kinetic fitting to approximately 592 µM in a 1:1 model (Fig. S1). This evidence confirmed the ability of this protein to bind physically the drug-like molecule in vitro. Comparing to the cognate ligand, ILK displays better affinity in terms of K_D_ towards ATP (3.64 µM), according to data reported in the literature [31]. This is not surprising as the ILK catalytic core has evolved, probably losing its catalytic activity, but those changes in the ILK sequence and its associated three-dimensional architecture does not impair nucleotide binding capability, which is comparable to other ATP-binding pseudokinases such as STRADα or TYK2 JH2 [32].

### Protein energy landscape exploration calculations point to the nucleotide cleft

Once direct binding to ILK had been confirmed, we decided to study the molecular recognition phenomenon. As mentioned previously, the lack of information concerning the molecular interaction between Cpd22, and ILK prompted us to study this question using biomolecular modeling techniques. Given the ability to bind ATP, the nucleotide-binding site (herein pseudo-active site) is by far the most promising druggable pocket in ILK, similarly to kinases. As such, and given the reported driven hypothesis of Cpd22 development, our computational studies were conducted using a refined model of the kinase domain of ILK (henceforth ILK) generated from the PDB entry 3KMW [33]. This crystal contains the kinase domain of ILK bound to the *N*-terminal domain of α-parvin, with the pseudo-active site filled by an ATP molecule and a Mg^2+^ cation.

Based on the aforementioned observations, the main hypothesis driving the computational work was that Cpd22 could be recognized by the pseudo-active site of ILK. To lay the groundwork for our subsequent calculations, we started searching for potential binding sites, considering the whole protein surface. To this end, we started by setting up several non-biased MD simulations to allow the ligand to diffuse and associate with ILK in the solvent. Thus, Cpd22 was randomly placed in the solvent around ILK and, after three independent replicas of 2 μs, the ligand was found not to reach any stable binding site on the protein (data not shown). Consequently, we decided to employ PELE (Protein Energy Landscape Surface), a relatively novel Monte Carlo algorithm, to investigate Cpd22 binding in a computationally affordable manner [34]. The PELE algorithm has been used successfully to determine the binding mode and location of several enzyme substrates, drugs and protein–protein inhibitors [35, 36]. For PELE calculations, seven independent simulations were carried out starting from four different starting ligand positions in order to explore the ILK surface (Fig. S2A).

This protocol allowed us to check and study the tendency of Cpd22 to associate with the ATP cleft or other regions on the protein. During these simulations, the ligand was protonated at the distal nitrogen atom of the piperazine ring given the estimated p*K*_a_ for a realistic physiological environment (pH 7.4). A refined ILK model was employed by extracting the ATP molecule but maintaining the metallic cation and by placing Cpd22 in up to four different locations around ILK (Fig. S2A).

**Fig. 3:**
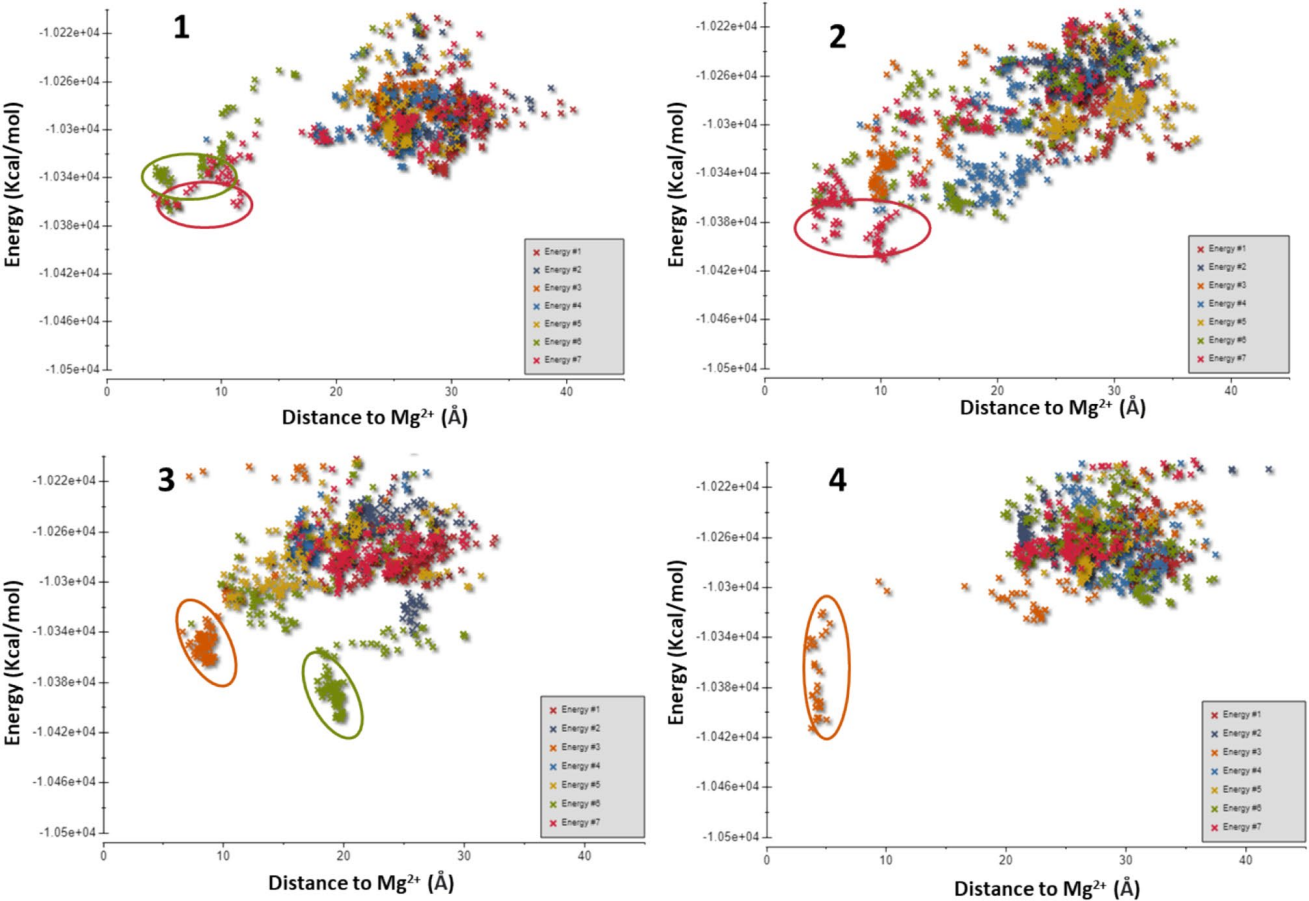
PELE simulation interaction energy plots versus distance to Mg^2+^, starting with the four poses generated manually. The scatter plots for the seven single-CPU simulations generated automatically by the PELE web server are shown in each panel. Circles denote the accepted Monte Carlo steps that have the lowest energy and are located near the ATP pocket

Global exploration of the ILK surface revealed various Cpd22 minimal energy locations with strong interaction energies closer to the Mg^2+^ atom (Fig. 3). Interestingly, these calculations showed that, at least one of the seven trajectories generated for each exploration simulation over the ILK surface reached the ATP pocket, and these tended to have the best energy values (Fig. 3 and S2B). Furthermore, in the case of starting positions 1 and 3, two independent trajectories found the pseudo-active site of the protein to be most favorable region for Cpd22 binding (see Fig. S2B). The remaining simulations did not converge on a consensus or well-defined site over ILK. Moreover, the resulting PELE interaction energies were worse than for the trajectories that reached the ATP cleft between the *N*- and *C*-terminal lobes. These findings suggest that Cpd22 tends to bind to a shallow cavity rather than a solvent exposed surface, as expected for a small molecule modulator that targets an enzymatic active site. Visual examination of the final protein–ligand complexes inside the ATP pocket showed that the resulting Cpd22 poses were not similar, thus resulting in different orientations of the molecule inside this cavity (Fig. S2B). This is not surprising as the PELE protocol chosen was designed for a global exploration of the protein rather than ligand pose refinement.

As the initial approach was intended to roughly identify a plausible location for Cpd22 binding, more sophisticated methods were needed to propose a plausible and accurate binding mode. In this regard, we first carried out docking calculations considering a rigid receptor and flexible ligand during the conformational sampling step. Glide, which is a broadly validated docking method, in XP mode was selected for docking calculations [37, 38]. Its empirical scoring function was calibrated using kinase inhibitors in the training dataset and, as a result, Glide seems to be a very appropriate docking tool for the purpose of this study with a kinase-like protein such as ILK.

As mentioned above, PELE simulations converged on the ATP binding pocket and adjacent areas, therefore docking calculations were performed in this region of the protein. Initially, the docking protocol was validated by redocking the ATP molecule as positive control (Fig. S3). This docking procedure was able to reproduce the ATP binding mode (Fig. S3B, − 21.1 kcal/mol).

**Fig. 4:**
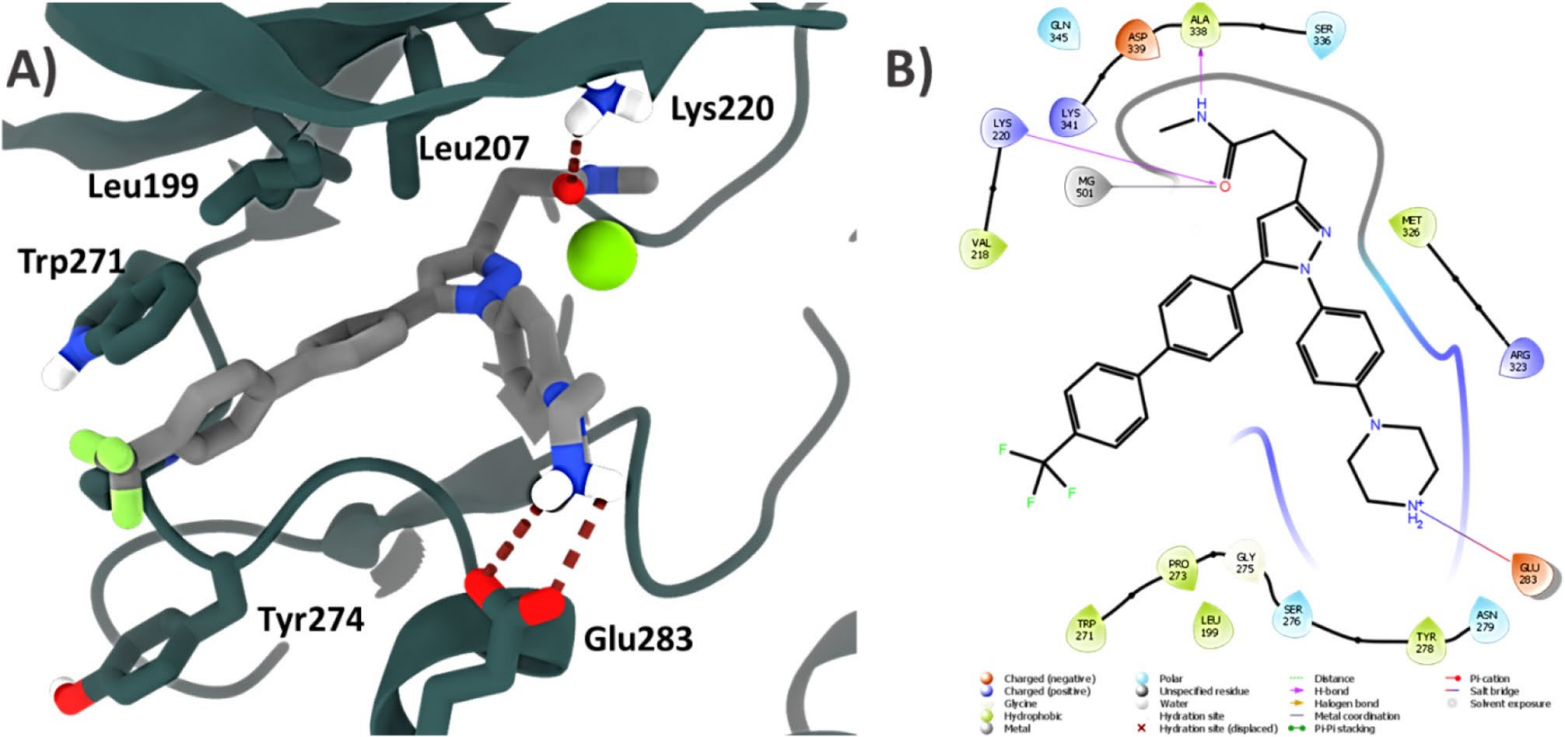
**A** Three-dimensional view of best docking pose predicted by Glide for Cpd22 in ILK. **B** Two-dimensional ligand-interaction diagram from docking pose generated with Maestro

Glide produced three different poses, which differed by less than 0.2 RMSD Å, thus resulting in a single well-defined binding mode (Fig. 4). Interestingly, the predicted binding mode was not one of those generated by PELE simulations. However, this was partly expected as the PELE global exploration was not intended to find an accurate binding mode. The Glide XP docking energies ranged from, − 6.1 to − 5 kcal/mol, thus supporting the plausibility of molecular recognition by the ILK pseudo-active site.

The molecule is accommodated inside the ATP pocket, close to the Mg^2+^ cation. Branch A of Cpd22 is oriented towards the outer region of the cleft, with the piperazine ring pointing towards Glu283. This facilitates an ionic interaction reinforced by a doubled hydrogen bond between the positively charged nitrogen atom of Cpd22 and the carboxylate group of Glu283 (Fig. 4). Given its strength, this is a key anchoring interaction and its presence also explains the experimental loss of activity when the piperazine ring is replaced by β-alanine or other heterocycles such as morpholine [29].

The second branch of the pyrazole ring, which corresponds to the *N*-methyl propanamide chain (branch B), is located in the region neighboring Mg^2+^. The oxygen atom from the carbonyl group is hydrogen bonded to Lys202 and might chelate the metal thanks to the lone electron pairs. A long-distance hydrogen bond (~ 4 Å) is observed between the Ala338 carbonyl and Cpd22–NH amide groups. With regard to the diaryl moiety positioned at C5 of the pyrazole ring (branch C), docking simulations show that it is oriented towards a hydrophobic tunnel delimited by the side chains of amino acids Leu199, Leu207, Trp271 and Pro273. A detailed visualization of the solvent-accessible surfaces revealed that this is a narrow space, thus hindering the accommodation of bulky groups such as the phenanthrene ring, as reported by Liu and collaborators [29].

Since ILK has been also crystallized in absence of Mg^2+^ cation (PDB ID: 3UMU) we also considered this scenario for docking purposes. In this case, Glide retrieved two completely different binding modes with docking energies of − 4.1 and − 1.6 kcal/mol respectively (Fig. S4). Overall, breakthroughs seem to indicate that Mg^2+^ plays an important role in Cpd22 orientation inside the pseudo-active site, since both poses thus obtained, were very different among them, as well as its docking scores compared to ILK-Mg^2+^ docking. Interestingly, none of docking orientations stablished the directional ionic bond reinforced by hydrogen bond between Glu283 and the protonated piperazine ring, explaining the lower docking scores obtained. In these binding modes Glu283 is not able to interact with Cpd22, which orients its branch A towards Trp271 (Fig. S4A) or to the hydrophobic sub-pocket (Fig. S4B). As such, predicted binding energies are lower compared to ILK-Mg^2+^ docking results and these poses could not account for experimental SAR. Moreover, predicted docking energies were also worse compared to ILK-Mg^2+^ models. Considering all of these results and recent findings which link ILK to ATP-Mg^2+^ bound as a trigger for ILK activity [39], we maintained the Mg^2+^ cation in our subsequent simulations.

### Molecular dynamics simulation studies

Based upon the results discussed above, our findings point to the pseudo-active site of ILK as being the target cavity for Cpd22 molecular recognition. Molecular simulations, especially all-atom molecular dynamics (MD) simulations, are an essential tool for the study of biomolecules in a time-dependent manner. In this regard, classical MD can provide a realistic picture, at an atomic level, of how molecular recognition between a protein and a ligand occurs. This permits a better understanding of the interaction between Cpd22 and ILK and its implication for the stability and conformational space explored by the protein–ligand complex.

Initially, we aimed to validate the binding mode generated by way of docking calculations. To this end, three replicas of a 1 μs classical MD with explicit water molecules were performed (ILK-Cpd22). To monitor ligand binding at the proposed site, we measured the root mean square deviation (RMSD) corresponding to the heavy atoms of Cpd22 as the observable variable, combined with a visual inspection of the protein–ligand trajectories. Furthermore, to compare and investigate the solution structure and flexibility of ILK in the absence of any ligand, an additional three MD simulations of 1 μs were carried out in explicit water with the protein alone (Apo-ILK).

The simulated systems were considered equilibrated beyond 100 ns, as shown by the RMSD convergence (Fig. S5). Furthermore, the protein remained stable during simulations with mean backbone RMSD values of approximately 2 Å. It is noteworthy that ILK-Cpd22 replicas presented lower RMSD values, thus suggesting stable and less flexible systems compared to the ILK kinase domain alone in solution. This observation seems to be related to the effect that Cpd22 can exert on the protein upon ligation.

**Fig. 5:**
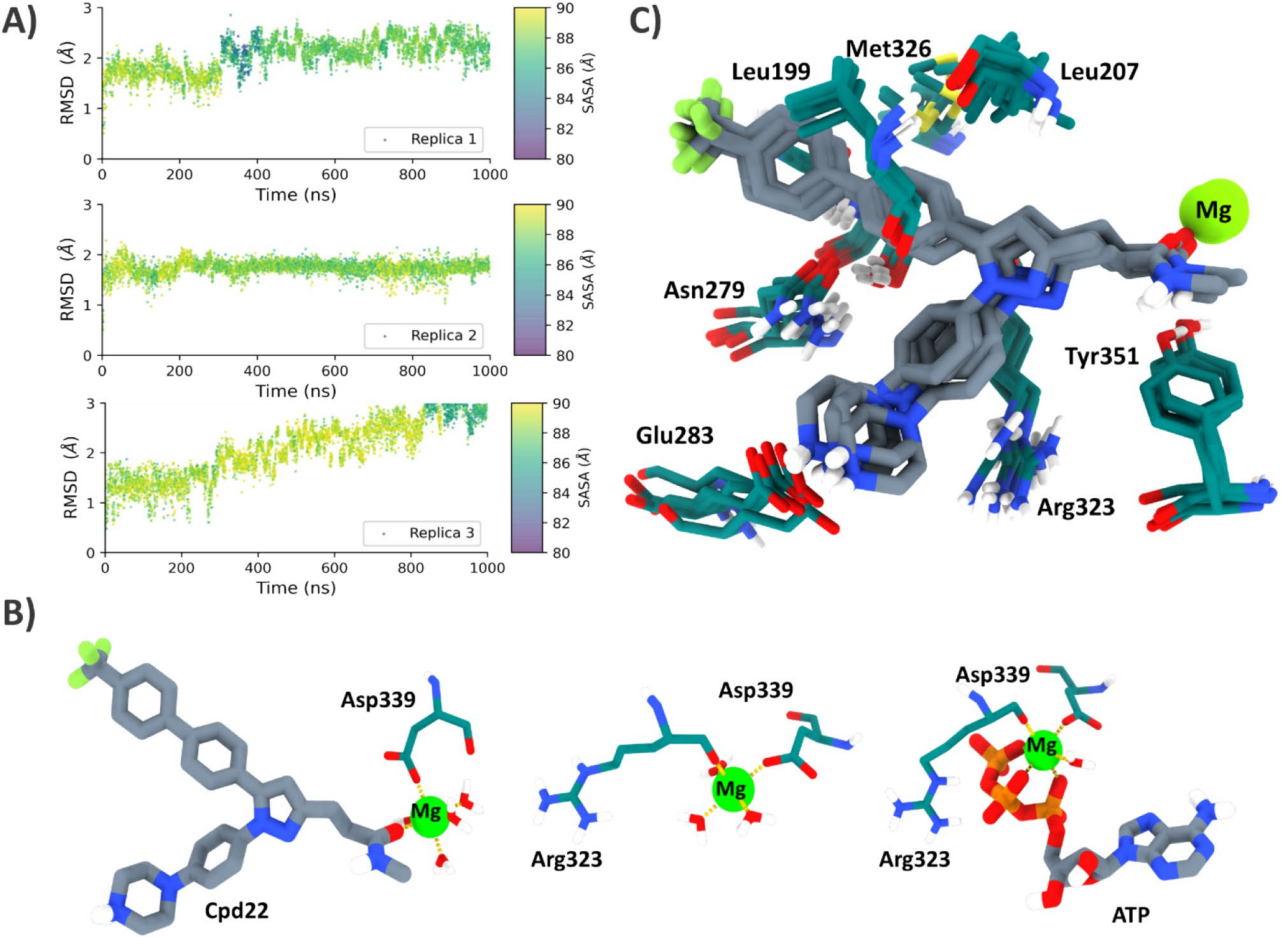
**A** Scatterplot showing evolution of the heavy atom root-mean-square deviation (RMSD) of Cpd22 computed for three trajectories. Points are colored according to the ligand SASA value in the snapshot. **B** Coordination sphere observed during MD simulation for ILK-Cpd22 (left), apo-ILK (center) and ILK-ATP (right) complexes. **C** Superimposition of the four minimized complexes obtained after the simulated annealing protocol. Close-up view of Cpd22 (sticks with **C** atoms colored in grey) lodged inside the ATP binding site of ILK (sticks with **C** atoms colored in dark cyan)

With regard to Cpd22, during our MD simulations, only one metastable binding mode was observed across the three replicas. This is reflected in terms of low fluctuating ligand RMSD values during the simulation, as shown in Fig. 5A and S5. Furthermore, ligand conformational changes are also associated with a slight decrease in the solvent accessible surface area (SASA) values during the majority of the simulation, which reflects the tendency of the ligand to bury in the ATP pocket. Cpd22 only started to abandon the active site during the third replica, after 800 ns, probably initiating an unbinding event that was not completed in the remaining simulation time.

Moreover, a detailed visual examination of the MD simulation allowed us to observe how the initial binding mode (from docking calculations) suffered subtle but significant shifts that altered some of the initial interaction patterns. Thus, a few nanoseconds after the production phase, Cpd22 is accommodated inside the ATP pocket. As result, branch B orients its carbonyl group towards the Mg^2+^, thus chelating the cation during the rest of the simulation by displacing a solvent water molecule. In the original crystal structure (PDB: 3KMW) [33], Mg^2+^ is coordinated by the carboxylate functionality of Asp339 and the coordination sphere is completed by three oxygens atoms from the α-, β- and γ-phosphate groups of ATP, plus two water molecules (Fig. S3B).

Interestingly, in our simulations for ILK bound to Cpd22, the carbonyl group from ligand branch B penetrates into the Mg^2+^ coordination sphere by way of the oxygen atom (substituting the role of ATP γ-phosphate). Therefore, in the ILK-ATP and apo-ILK trajectories, the Asp339 carboxylate and Arg323 carbonyl participates in the coordination with the cation (Fig. 5B). Finally, four water molecules for apo-ILK and ILK-Cpd22 and only one molecule in the case of ILK-ATP are in charge of maintaining a quasi-octahedral coordination geometry across the simulations (Fig. 5B). Despite the unclear catalytic ability of ILK [14, 31], we observed that coordination waters remain stable during our MD simulations, filling the Mg^2+^ coordination sphere instead of other ILK residues around the vicinity of this pocket. This observation seems to indicate that probably Mg^2+^ is not tightly bound to ILK and, and probably Cpd22 also plays an active role to chelate Mg^2^ and thereby preventing its loss from the pseudo-active site as previously reported for ATP [39].

Ligand rearrangement is also accompanied by a partial reorientation of the biphenyl moiety in the hydrophobic pocket to an outer region. This sub-pocket, which was previously occupied by branch C, corresponds to the location where the adenine ring of the ATP molecule is lodged (Fig. S6) and is a critical optimization region for kinase inhibitors. In light of these findings, it appears that the incorporation of a scaffold on Cpd22 that can mimic hydrogen bonding features will be beneficial for ligand enthalpic optimization [40, 41], a strategy that was not fully covered by the inhibitors used in the original study [29]. Moreover, the pyrazole ring is partially shifted, thus meaning that it can occasionally establish a new hydrogen bond between the pyrazole N2 and Arg323 sidechain (mean 10.58% lifetime occupancy across the three replicas).

Overall, these observations seem to indicate that the Cpd22 binding mode is mainly governed by the strong ionic interaction between the piperazine ring and Glu283 (which does not suffer significant changes). Additionally, its orientation is also stabilized by the stable coordination bond with the Mg^2+^ cation, which also remained quite stable during MD simulations, with a mean distance between the oxygen and magnesium atom pairs of around 2 Å across replicas (Fig. S7).

To further understand and support this proposal of binding mode, a simulated annealing protocol was carried out to characterize this protein–ligand conformation as a local or even global minimum in the conformational landscape. Simulated annealing techniques have previously been employed successfully to optimize structures from experimental assays [42, 43], characterize binding modes [44], and predict protein conformations [45, 46]. Using this protocol, it was discovered that the Arg323 sidechain changes its orientation towards the phenyl ring present in Cpd22 (branch A) for all ILK-Cpd22 complexes (Fig. 5C). As such, the cationic amino acid receives π density from the phenyl ring, thus contributing to Cpd22 anchoring. In terms of ligand optimization, this interaction can be improved by incorporating more electron rich aryls (like a bioisosteric replacement of the benzene by tiophene) in branch A. In addition, the *N*-methyl substituent (branch B) is able to establish a CH···π interaction with the Tyr351 aromatic ring. This finding may explain the experimental evidence that replacement of a methyl by an ethyl group preserves the desired activity [29]. Similarly, further optimizations substitution of methyl and alkyl groups in the amide by aromatic rings could have also a good impact in ligand binding due to π-stacking interactions with Tur351.

### Cpd22 binding induces changes in ILK structural plasticity

**Fig. 6:**
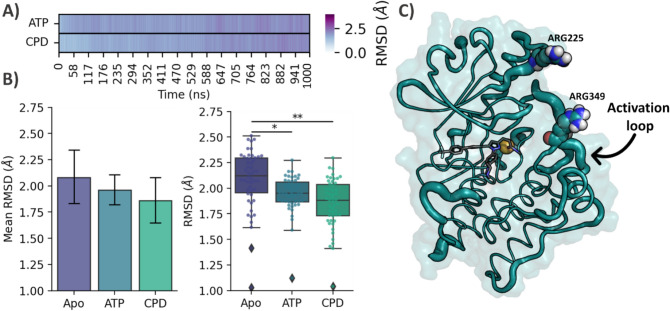
**A** Heatmap plot of mean ligand heavy atom RMSD values averaged for three independent replicas. **B** Bar and whisker plot of trajectory median backbone RMSD values for apo-ILK, ILK-ATP and ILK-Cpd22 (n = 3 trajectories, **p < 0.005 and *p < 0.05, t-test compared to apo-ILK). **C** Cartoon “putty” representation of ILK-Cpd22 showing regions with high RMSF values as thick cartoons

In light of the findings discussed above, we then studied whether Cpd22 binding can induce conformational changes to the ILK kinase domain. To this end, an extra complex corresponding to ILK bound to its cognate ligand ATP (ILK-ATP) was simulated in triplicate. This simulation also displayed a stable profile in terms of RMSD, especially for ATP (Fig. S4), the hydrogen bonding network for which anchors the molecule strongly during simulations. When comparing stability between ATP and Cpd22 molecules, we found that the former is better accommodated inside the pseudo-active site of ILK in terms of mean RMSD (1.64 ± 0.09 Å) than the synthetic ligand (1.95 ± 0.28 Å) (Fig. 6A). This is not surprising as the pseudo-active site conserves most of the molecular determinants to bind nucleotides, with ATP being the natural ligand of this pseudokinase [31].

We then focused on the influence that ligand binding could have on the molecular rigidity of ILK. An exhaustive RMSD analysis led us to conclude that the overall flexibility of the protein is significantly higher in the absence of any ligand (apo-ILK) compared with both holo proteins (Fig. 6B). In terms of RMSD, apo-ILK exhibited a mean value of 2.08 ± 0.25 Å (SD) while ILK-ATP showed a slightly lower value (1.96 ± 0.1 Å). This is in good agreement with recent studies relating ATP binding to a conformational stabilization of the ILK kinase domain, thus also validating our approach [47]. Moreover, our observation is in full agreement with experimental studies, which observed stabilization induced by ATP binding rather than gross conformational changes [7, 31, 47].

A similar picture was observed when comparing ILK-Cpd22, which resulted in an even more pronounced reduction in the mean RMSD value (1.86 ± 0.21 Å) when compared to ILK-apo and ILK-ATP. Indeed, this was also corroborated by the change in protein radius of gyration, the mean values of which decreased across the holo systems (see supporting info Table S1), thus indicating that ILK stabilization by pseudo-active-site ligands is accompanied by a slight increment in protein compactness. This effect may be of interest from the point of view of ILK protein–protein interactions with PINCH and parvin partner adaptor proteins, as the response elicited by Cpd22 on ILK flexibility as a whole could affect PPI assembly and/or stability given the lower conformational motions. This hypothesis is supported by the previously observed effect of ATP binding, which stabilizes the pseudokinase domain, thus facilitating a more effective interaction between the focal adhesion proteins PINCH and parvin [39, 47].

To study the flexibility in terms of single residues, RMSF values per Cα were calculated (supporting info Fig. S8) and inspected visually in the context of protein dynamics. Fluctuations in the nucleotide-binding cleft residues were observed in the case of ILK-Cpd22 simulations, as would be expected due to ligand accommodation inside the pocket, along with branch C shifting alongside the hinge region. Remarkably, we observed differences in the pseudo-active site loop, spanning residues 339 to 359. This segment contains the Arg349 residue which is implicated in an allosteric network connecting ILK kinase-like domain to α-parvin via the activation loop (Fig. 6C) [47]. While for apo-ILK this segment remains with low mobility (according to RMSF values, Fig. S8), Cpd22 and ATP induced a significant increase (up to approx. 6 Å for the latter) in this region. Interestingly, for ILK-Cpd22 simulations, higher RMSF values were also observed in a second segment spanning residues His309 to Asn321, which is located in the C-lobe subdomain and flanking the pseudo-active binding site. Another less intense peak, corresponding to the glycine-rich P loop (residues 202–205), fluctuates to accommodate ATP and Cpd22 inside the cleft with values close to 2 Å (Fig. S8).

Because of the higher mobility observed for the activation loop during our simulations, a close inspection of those residues implicated in the allosteric network with α-parvin was carried out raising some interesting breakthroughs (Table 1).

**Table 1:** Calculated root-mean-square fluctuation (RMSF, Å) values for Arg225 and Arg349 obtained from 1 µs MD simulations (SD = Standard deviation)

	Replica 1	Replica 2	Replica 3	Mean	SD
Apo-ILK					
ARG 225	1.57	1.57	1.56	1.57	0.004
ARG 349	1.81	1.81	1.62	1.75	0.09
ILK-ATP					
ARG 225	1.8	1.83	2.53	2.06	0.33
ARG 349	2.79	2.79	5.45	3.68	1.25
ILK-CPD22					
ARG 225	1.66	1.66	2.37	1.90	0.33
ARG 349	3.49	3.49	1.79	2.92	0.79

The pseudo-active site of ILK seems to be implicated in an allosteric network towards α-parvin in which ATP molecule plays a major role [47]. Communication over the *N*-lobe from the kinase-like domain of ILK is mediated by two arginine residues (Arg225 and Arg349) close to the ILK:α-parvin interface (Fig. 6C). They form salt bridges with Glu332 and Asp336 in α-parvin, and their abolition in single and double mutants leads to a reduced α-parvin binding compared to the ILK wild type [47]. In our apo-ILK simulations these amino acids preserve some residual mobility along the whole dynamics. In contrast, the presence of an ATP molecule increases their mobility, especially for the Arg349 (Table 1), as previously observed and reported for the ILK-ATP complex without α-parvin [47]. Notoriously, this effect is also observed in Cpd22 bound systems, suggesting the ability of this molecule to trigger the allosteric network towards α-parvin interaction region. Overall, these data suggest that the effect of Cpd22 over the ILK pseudo-kinase domain can be comparable to those induced by the ATP nucleotide, increasing the mechanical stability of the ILK:α-parvin complex [47].

### Analysis of end-point binding free energy

To gain further support for our binding mode hypothesis and further insights into binding thermodynamics, the approximate free energy of binding was calculated using the MM-ISMSA method [48]. In addition, this method was also employed for the ATP molecule in order to have a positive control as reference (Supporting Table S2). The ligand binding energy calculations obtained using this method are easily reproducible and computationally inexpensive compared to other more sophisticated methods, and provide good accuracy [44, 48–50].

The free energies averaged across replicas revealed how both ligands can bind in a thermodynamic favorable context to the nucleotide pocket of ILK with mean values of − 50.49 and − 48.93 kcal/mol for ATP and Cpd22 respectively. Our calculations revealed that the predicted interaction affinity for ATP towards ILK is slightly more favorable than for Cpd22 (Table S2), which is expected as the former is the cognate ligand of this pseudo-kinase.

**Fig. 7:**
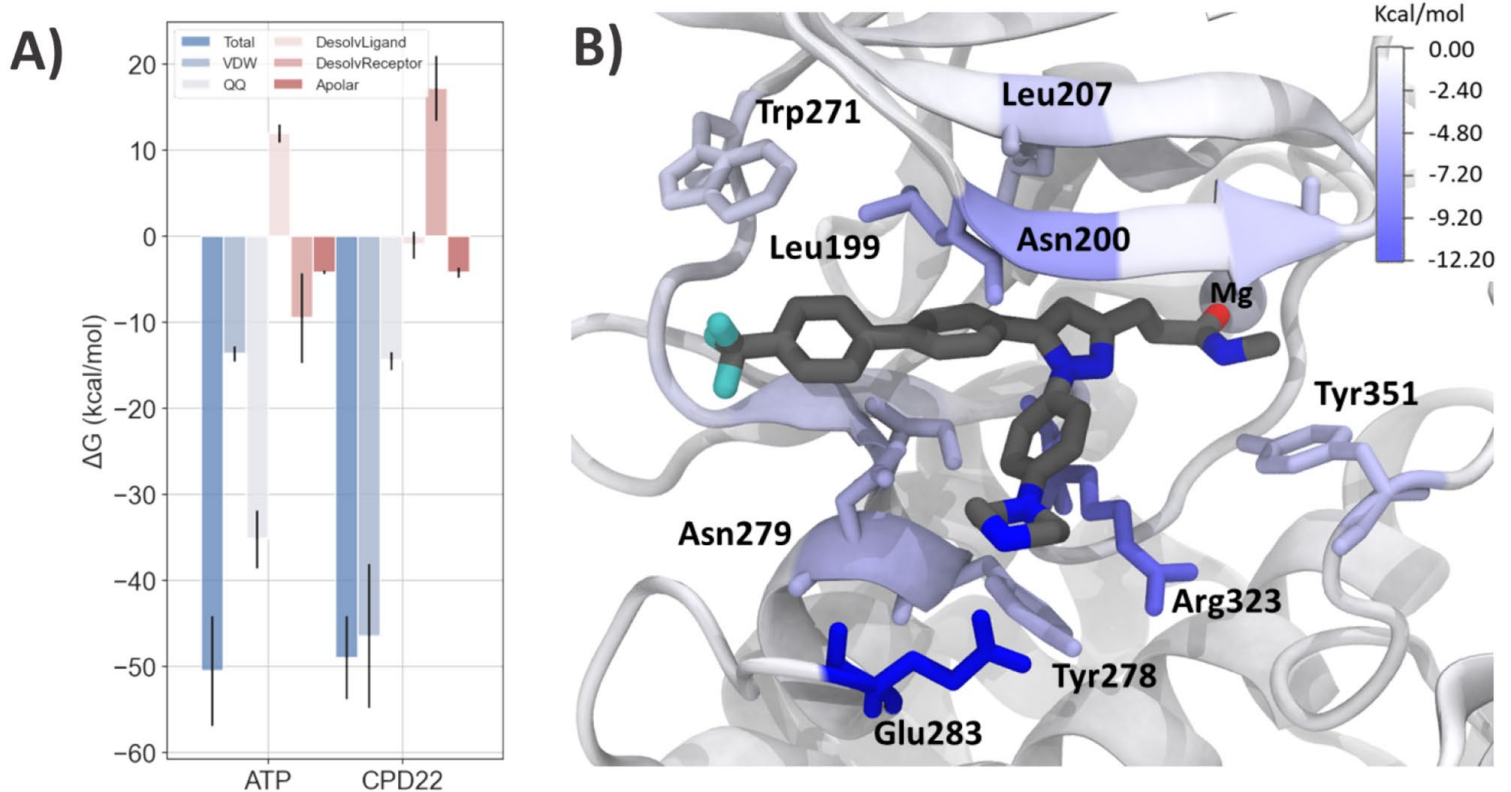
**A** Calculated total interaction energies (dark blue, kcal mol^−1^) and energy contributions for the binding of ATP and Cpd22. The averages (± standard deviation) were calculated from the last 500 ns (ensemble of 2500 snapshots) across three independent replicas. **B** Representative snapshot from a replica of the MD simulation used to show free energy decomposition per residue. The residues are colored to represent their total free energy component of the MM-ISMSA free energy method, with blue values indicating stabilization of the ligand

In addition, a detailed view on the MM-ISMSA components to the free energy raise interesting differences. Indeed, whereas the main favorable contribution for ATP binding energy is provided by electrostatic interactions (coulombic term), in the case of Cpd22 it is given by van der Waals forces (12-6 Lennard–Jones potential) (Fig. 7A). This can be explained by the larger number of polar substituents present in the ATP molecule, especially the three phosphate groups. These moieties participate actively in the interaction with several polar and charged residues of the binding pocket, including Ser204, Lys220, Asn279, Arg323, Asp339, and Lys341, thereby exerting a strong influence on the binding pose and its stability during MD simulations (Fig. S5B). Additionally, higher desolvation penalty is observed for the ATP molecule compared to CPD22, which is explainable for the presence of charged phosphate groups as well as a polar ribose ring which must be highly solvated by water molecules in the unbound state. This suggest that CPD22 presents a good balance between polar and non-polar groups, and further improvements for binding potency can be accounted by apolar groups with low desolvation penalties. A detailed analysis of the per-residue contribution to the binding free energy for CPD22 may reveal the importance of some residues and their role in anchoring the molecule into the pseudo-active site cleft (Fig. 7B and S7). As observed previously in docking and MD simulations, the largest contribution comes from Glu283 (approx. − 10 kcal/mol averaged over three independent replicas) and its ionic interactions with the piperazine moiety (Fig. 7B). Interestingly, Arg323 is also considered to be an important residue for the binding free energy according to these calculations, although it depends on the side-chain orientation. During MD simulations, Arg323 can flip and accommodate its sidechain to establish a cation**···**π interaction between the guanidinium group and the ligand phenyl ring from branch A. Furthermore, Arg323 also interacts with this branch of Cpd22 via van der Waals interactions with the CH_2_ groups in its side chain, thus making this residue an important anchoring partner and stabilizing branch A.

Further contributions are provided by aliphatic and aromatic amino acids such as Leu199, Leu207, Tyr278, Trp271 and Tyr351, mainly because of non-polar contacts with Cpd22. It is worth noting that the stable coordination observed during MD simulations influences the binding free energy via the Mg^2+^ cation contribution (approx. 2 kcal/mol across replicas) to Cpd22 binding.

### Conclusions

Since its discovery more than 20 years ago, ILK has become a very interesting and fascinating protein with controversial kinase activity but a well-established scaffolding function in the dynamic of focal adhesions. Its initial validation as a promising target for the treatment of neoplastic disease prompted the efforts of pharmaceutical and biotechnology companies to find potential inhibitors for human therapies. The small molecule Cpd22 was discovered to be a promising candidate for clinical development. However, there was no clear evidence about ILK modulation by physical binding of Cpd22 to the protein neither the detailed mechanism of molecular recognition. As a result of SPR measurements and the different molecular modeling techniques used herein, we have identified and confirmed that this molecule binds to ILK via its ATP pocket with affinity and a similar behavior to the cognate ligand. This phenomenon leads to subtle structural changes in the ILK kinase domain with a similar tendency to ATP. In this regard, our breakthroughs suggests that the mechanism of action for Cpd22 implicates the stabilization of the pseudo-kinase domain which could eventually affect to the scaffolding properties of the ILK linked to the cytoskeleton rather than active site catalysis. Our studies also explain the experimentally observed SAR and validate our results and the protein–ligand interaction model. These data allow us to explain experimental breakthroughs, thereby paving the way to the design of more potent and sophisticated inhibitors. In this regard, pseudo-active site targeting ligands should also optimize ionic interactions with Glu283 as important anchoring residue. Additionally, our results may indicate how, despite the unclear catalytic capacity of ILK and the role of the magnesium atom present in the pseudo-active site, this can act as an anchoring point for the molecular recognition and binding of drug-like molecules or chemical probes. As such ligand design should consider cation chelation as a strategy for ILK binding and modulation as well as the strong ionic interaction with Glu283.

## Material and methods

### Protein preparation

Refined molecular models of ILK were generated starting from the crystal structure of the ILK kinase domain bound to the CH2 α-parvin domain (PDB ID: 3KMW) [33]. Chain A (comprising residues His185 to Lys452 from ILK) was retrieved from the web and then refined using the Protein Preparation Wizard module implemented in Maestro 2020–2022 (Schrödinger LLC, New York) by adding hydrogens and setting tautomeric and protonation states at pH 7.26 using the default protocol. To maintain electroneutrality in protein terminations, the N-terminus was capped with an ACE group and the C-terminus with an NME group. The apo-form was generated by extracting the ATP molecule from the ILK binding site manually using PyMOL (The PyMOL Molecular Graphics System, Version 2.1 Schrödinger, LLC).

### Ligand preparation

The Marvin Sketch suite 21.3.0 (ChemAxon, http://www.chemaxon.com/) was used to generate a 2D representation of Cpd22 and to investigate the predicted p*K*_a_ value for the piperazine ring (p*K*_a_ = 8.9) in order to establish the protonation state under physiological conditions. The molecule was then exported to Maestro 2020–2022 in sdf format to generate an appropriate 3D structure using the LigPrep module. This tool was used to obtain a minimized geometry and to assign partial atom charges according to the OPLS3 forcefield for docking calculations [51].

### PELE global exploration simulations

Protein Energy Landscape Exploration (PELE) is a Monte Carlo technique that includes protein structure prediction, thus allowing the exploration of potential binding sites via several sequential steps: ligand and/or protein perturbation followed by side rotamer sampling and energy minimization [34]. PELE has been used to find the small molecule binding site in proteins and to provide a dynamic perspective of substrate binding [35, 36]. In the case of ILK, in order to determine the Cpd22 binding site, four simulations corresponding to different starting geometries were generated by placing the molecule over the refined ILK-apo surface manually, at a distance of approximately 5 Å, using UCSF Chimera 1.15 [52]. Each starting position was submitted to the PELE simulation webserver (https://pele.bsc.es/pele.wt) and run in the MareNostrum IV cluster from the Barcelona Supercomputing Center with seven cores, each core performing 10,400 total PELE Monte Carlo steps. Protein and ligand parameters were derived from the OPLS2005 force field, with energies provided in kcal/mol. The main variables studied during these simulations were the protein–ligand interaction energies, the SASA of the ligand, and the distance between the Mg^2+^ atom coordinates and the Cpd22 center of mass. The PELE webserver tools were used to generate all energy plots [53].

### Ligand docking

The starting conformation of the ligand within the ILK ATP pocket was determined by docking. Glide software [37], in XP mode [38], was chosen to perform flexible docking calculations. Prior to docking simulations, a three-dimensional grid containing receptor properties and information was generated, with its centroid being located into the ATP pocket of the previously refined ILK structure. The inner grid box was limited to a cube with an edge of 11 Å. During docking, ligand sampling was set to flexible, and the protein kept rigid, with the maximum number of poses set to 10 and the rest of the parameters as default. Docking protocol was validated by redocking the ATP molecule inside the ILK model (RMSD = 1.17 Å).

### Molecular dynamics simulations

MD simulations were performed for three different systems, namely ILK-apo, ILK-ATP and ILK-CPD22, using the python software OpenMM 7.2.6 [54]. The protein and ATP parameters for the MD simulation were derived using the ff14SB force field [55]. The small molecule (Cpd22) was described using GAFF2 and partial atomic charges derived using the AM1-BCC [56] scheme implemented in antechamber, a module included in AmberTools19 [57]. *N*- and *C*-protein termini were capped with an acetyl (ACE) and a N-methyl-amide group respectively (NME) in order to avoid charged termini artifacts. Both the protein and protein–ligand complexes were immersed in a truncated octahedral box of TIP3P water molecules, which was extended 8 Å from the protein limits [58]. Additionally, the charge of the three systems was stabilized using monovalent ions (Na^+^ and Cl^−^) to reach electroneutrality. In order to apply a 4 fs time step during MD simulation, the hydrogen mass partitioning scheme was applied via parmed to modify the prmtop files, according to the protocol described on the Amber website (https://ambermd.org/tutorials/basic/tutorial12/index.php) [59]. A radius of 8 Å was chosen for nonbonded long-range interactions, and the PME method was used for electrostatic interactions, with a cut-off of 10 Å [60]. The Andersen thermostat at 298 K and the Monte Carlo barostat (1 atm) were applied during MD production under constant pressure and temperature (NPT). First, an initial energy minimization of the system during 6000 steps of the L-BFGS algorithm was performed [61], followed by an NVT equilibration for 5 ns. Thereafter, in order to increase the statistics of all parameters studied, three independent 1 μs production simulations were carried out for each system. All MD simulations were performed using the OpenCl platform in single-precision mode on a Windows 10 Workstation coupled to a Nvidia® Gigabyte GeForce RTX 2060 GPU unit.

The ultrafast cMM-ISMSA software was selected to calculate the binding free energy between ligands and protein [48]. This program implements a solvent-corrected binding free energy approximation based on a 12-6 Lennard–Jones potential and an electrostatic term (Coulombic) based on an implicit solvent model (IMS) with a sigmoidal distance-dependent dielectric function to model solvent effects on binding partners [62]. The use of free energy decompositions of the cMM-ISMSA binding free energy for annotating and visualizing molecular dynamics trajectories facilitates a per-residue analysis of results. To perform these calculations, the last 500 ns of each simulation (corresponding to the equilibrated systems) were saved as an mdcrd trajectory of 2500 snapshots using MDtraj [63].

Simulated annealing simulations were also performed using OpenMM software for the ILK-Cpd22 complex. After clustering the ILK-Cpd22 trajectory via k-means algorithm as implemented in cpptraj [64], a representative snapshot of the most populated cluster was selected as the starting Cartesian coordinates for the simulated annealing protocol. Four cycles, starting at 313 K and cooling the system to 273 K, were run in 1 ns, followed by 1000 steps of L-BFGS energy minimization for the “frozen” complex. The system was then warmed up to 313 K in 1 ns and equilibrated for 500 ps prior to reinitializing the annealing cycle.

To examine the convergence behavior of classical MD simulations, the root mean square deviation (RMSD) was calculated for protein backbone atoms (Cα, NH, O) using all snapshots. All analyses were carried out after aligning and imaging the whole trajectory to the starting frame with MDtraj [63] and MDAnalysis [65] suites. Statistical analysis of measurements was performed using the SciPy (1.6.1) library and a t-test or one-way ANOVA to test for significant differences. Graphics and plots were generated using Matplotlib [66] (3.3.2) and Seaborn (0.11.1) [67]. Trajectory visualization and image rendering were performed using VMD [68] and Chimera X 1.2 [69].

### Surface plasmon resonance

SPR experiments were performed at 20ºC using a Biacore X-100 HBS-EP apparatus (Biacore, GE) with 2% DMSO (running buffer) at 25ºC. ILK (Randox Life Sciences, 59.92 kDa) was immobilized on a CM5 sensor chip (Biacore, GE) following the standard amine coupling method. The carboxymethyl dextran surface of flow cell 2 was activated with a 7-min injection of a 1:1 ratio of 0.4 M EDC and 0.1 M NHS. The protein was coupled to the surface with a 7-min injection at 10 µg/ml in 10 mM sodium acetate, pH 5.0. The unreacted *N*-hydroxysuccinimide esters were quenched by a 7 min injection of 0.1 M ethanolamine-HCl (pH 8.0). The level of inmobilization was in the range of 5000 RUs. Flow cell 1 was treated in exactly the same manner as flow cell 2 (amine coupling procedure), but with no protein, for use as a reference. Prior to use 10 mM stock solutions of Cpd22 (from Calbiochem) were diluted several times until a final concentration of 5–50 µM in the running buffer. Typically, Cpd22 was injected onto the sensor chip a flow rate of 90 µL/min for a period of 1 min followed by a dissociation period of 1 min. After the dissociation process, an extra wash treatment was performed over the flow cells with a 50% DMSO solution. No regeneration was needed. Sensogram data were double-referenced, solvent-corrected and analyzed using the Biaevaluation X-100 software (Biacore, GE). A two-state reaction model was used to fit the experimental data.

## Supplementary Information

Below is the link to the electronic supplementary material.

Supplementary file1 (DOCX 4173 kb)

## Data Availability

The datasets and trajectories generated during and/or analysed during the current study are available from the corresponding author on reasonable request. Simulations were performed using the open-source molecular dynamics package OpenMM (https://openmm.org/) and systems were prepared using Ambertools19 (http://ambermd.org/AmberTools.php). All analyses were conducted with MDTraj (https://www.mdtraj.org/1.9.8.dev0/index.html) and MDanalysis (https://www.mdanalysis.org/) software via their distributions in Anaconda (https://www.anaconda.com/). For end-point free energy calculations, cMM-ISMSA software was compiled from source (https://github.com/accsc/cMMISMSA) to use in a Windows 64-bit machine. Data visualization was carried out with ChimeraX 1.3 (https://www.cgl.ucsf.edu/chimerax/) and VMD 1.9.3 (https://www.ks.uiuc.edu/Research/vmd/).

## Abbreviations

ILK: Integrin linked kinase
PDB: Protein data bank
RMSD: Root-mean square deviation
RMSF: Root mean-square fluctuations
