# Supplementary material for: Insight into the mechanism of molecular recognition between human Integrin-Linked Kinase and Cpd22 and its implication at atomic level
Source: J Comput Aided Mol Des. 2022 Jul 23;36(8):575–89. doi: 10.1007/s10822-022-00466-1 (PMC9512720; doi:10.1007/s10822-022-00466-1)
Supplement: Supplementary file 1 — Supplementary file1 (DOCX 4173 kb) [file 10822_2022_466_MOESM1_ESM.docx]

**Insight into the mechanism of molecular recognition between human Integrin-Linked Kinase and Cpd22 and its implication at atomic level**

*Javier García-Marín, ^a , b^ ^†,^* Diego Rodríguez-Puyol,^c ,d^ Juan J. Vaquero. ^a^*

^a^ Departamento de Química Orgánica y Química Inorgánica, Instituto de Investigación Química Andrés M. del Río (IQAR), Universidad de Alcalá (IRYCIS), Alcalá de Henares 28805, Madrid, Spain

^b^ Departamento de Química Biológica y Estructural, Centro de Investigaciones Biológicas, CIB-CSIC, C/Ramiro de Maeztu 9, 28040, Madrid, Spain.

^c^ Departamento de Medicina, Universidad de Alcalá (IRYCIS), Sección de Nefrología; Hospital Príncipe de Asturias, Alcalá de Henares 28805, Madrid (Spain)

^d^ Fundación Renal Iñigo Álvarez de Toledo (FRIAT) y Instituto de Salud Carlos III (REDinREN), Madrid, (Spain)


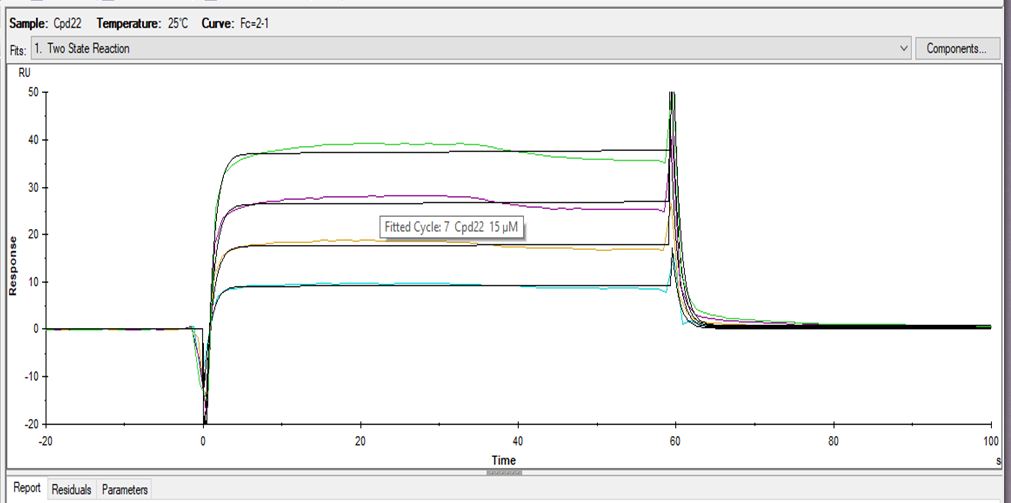


**Fig. S 1** Sensogram with the kinetic fitting of Cpd22 affinity constant (K_D_).


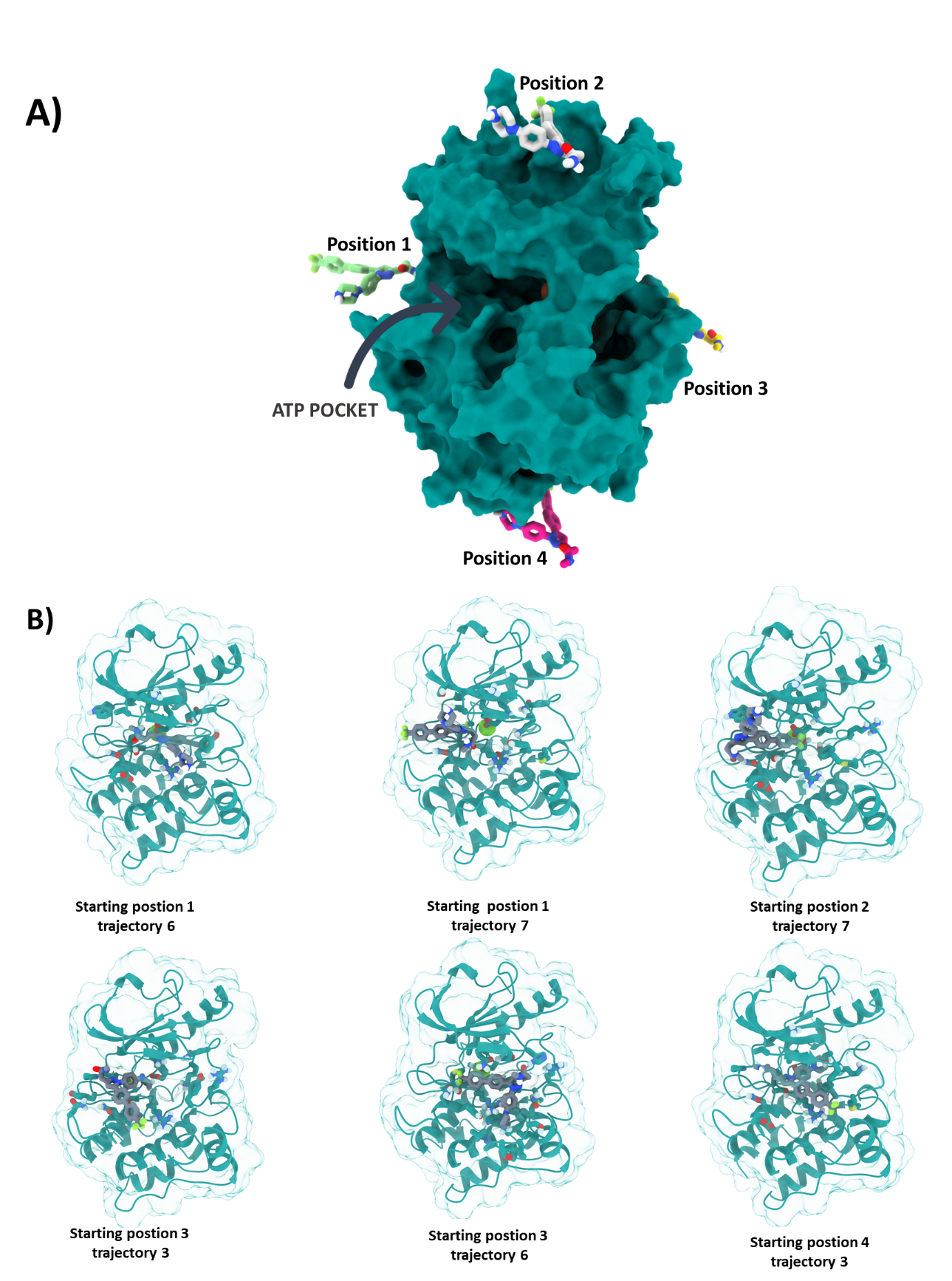


**Fig. S 2** **A)** Around the whole ILK surface binding site exploration, four initial Cpd22 colored sticks) starting points placed manually for binding site exploration. **B)** Last frames from PELE trajectories where Cpd22 reached the ATP cleft of ILK, which also corresponds to the most favorable in terms of calculated free energy.


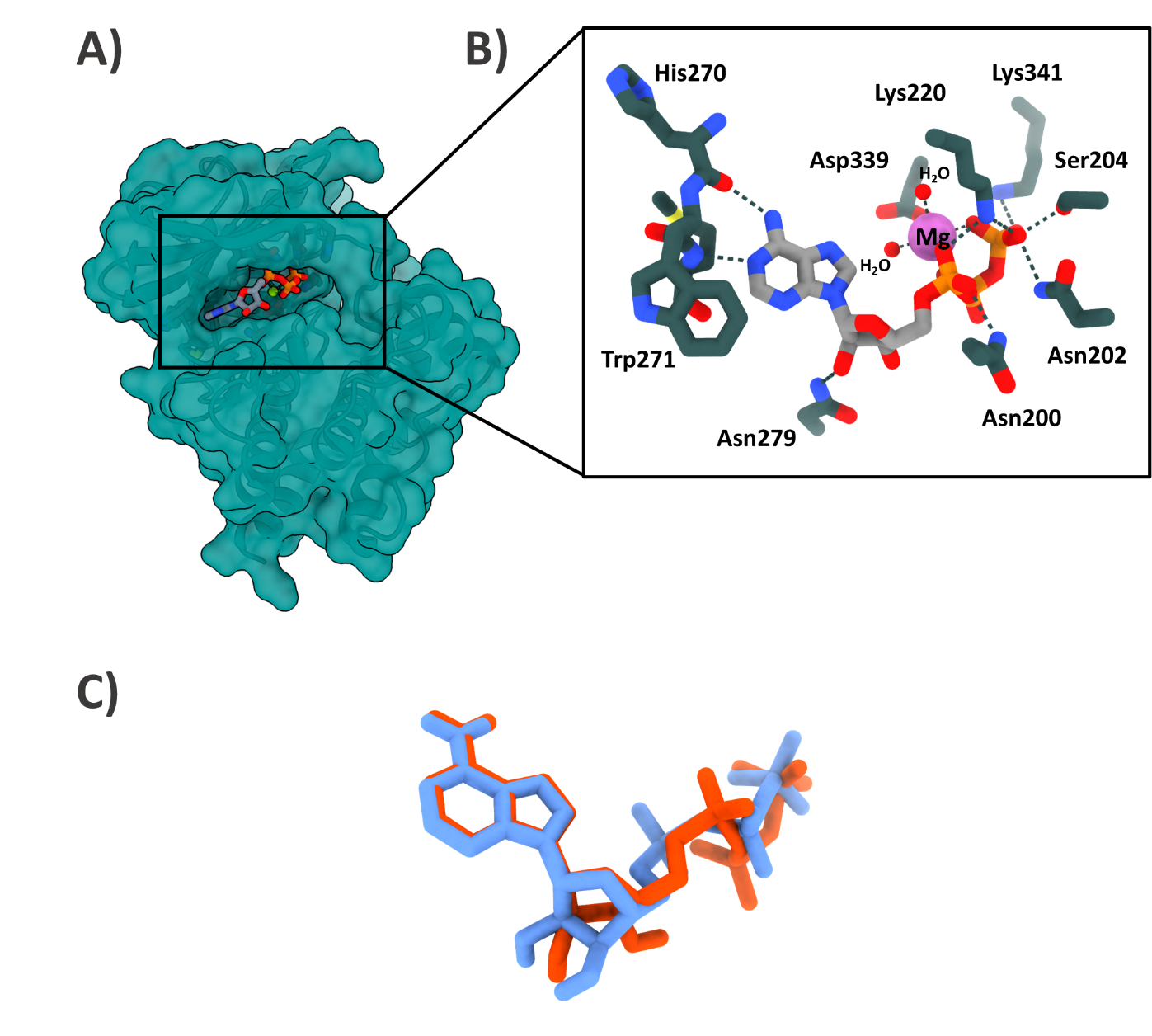


**Fig. S 3** A) X-ray crystal structure of the ILK kinase-like domain bound to ATP without α-parvin (PFB ID: 3KMW). B) Close view of the ATP molecule in the pseudo-active site and neighboring residues. C) Redocking of ATP molecule. In blue sticks the crystallographic conformation of ATP (PDB: 3KMW) superimposed to the obtained docking pose (orange sticks) by Glide XP.


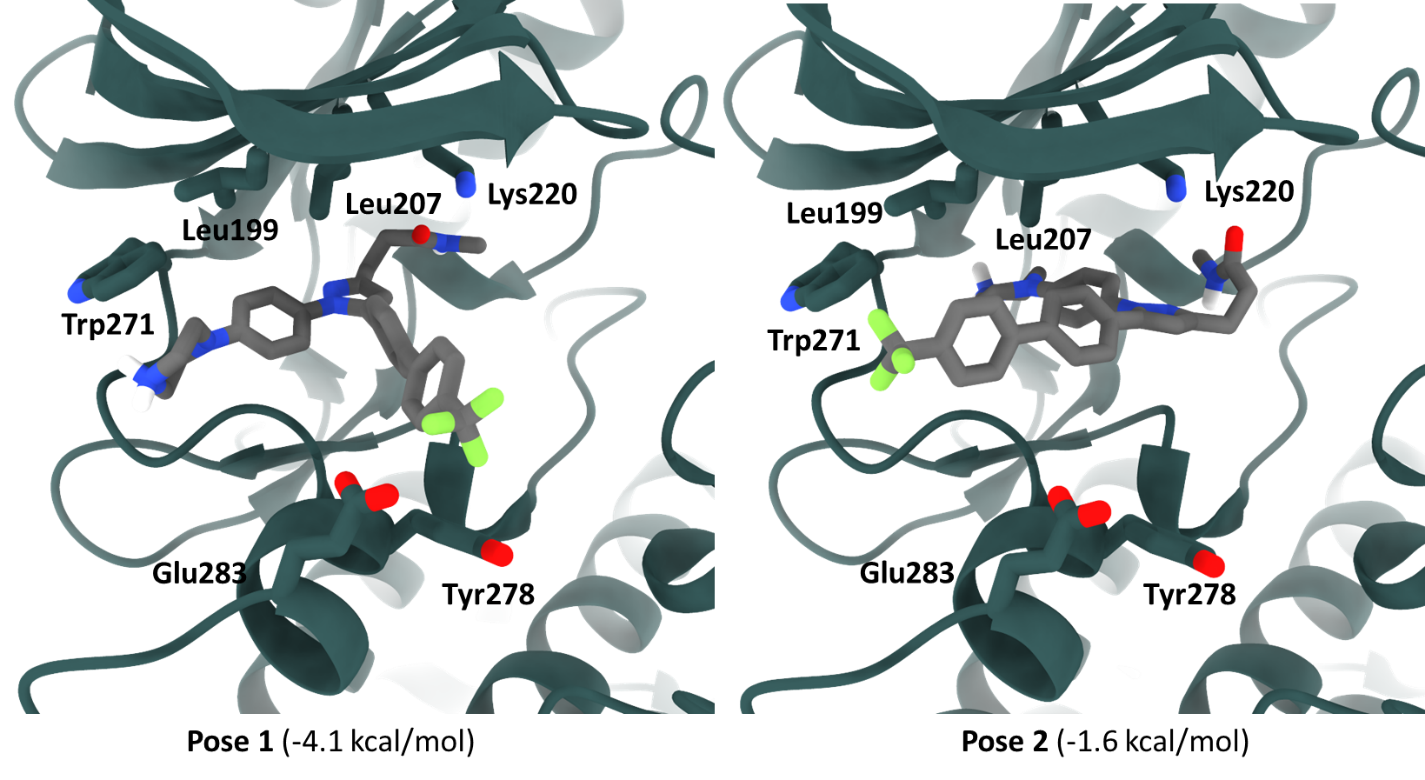


**Figure S 4.** Docking poses and scores obtained with Glide XP for Cpd22 into the ILK pseudo-active site without a Mg^2+^ cation.


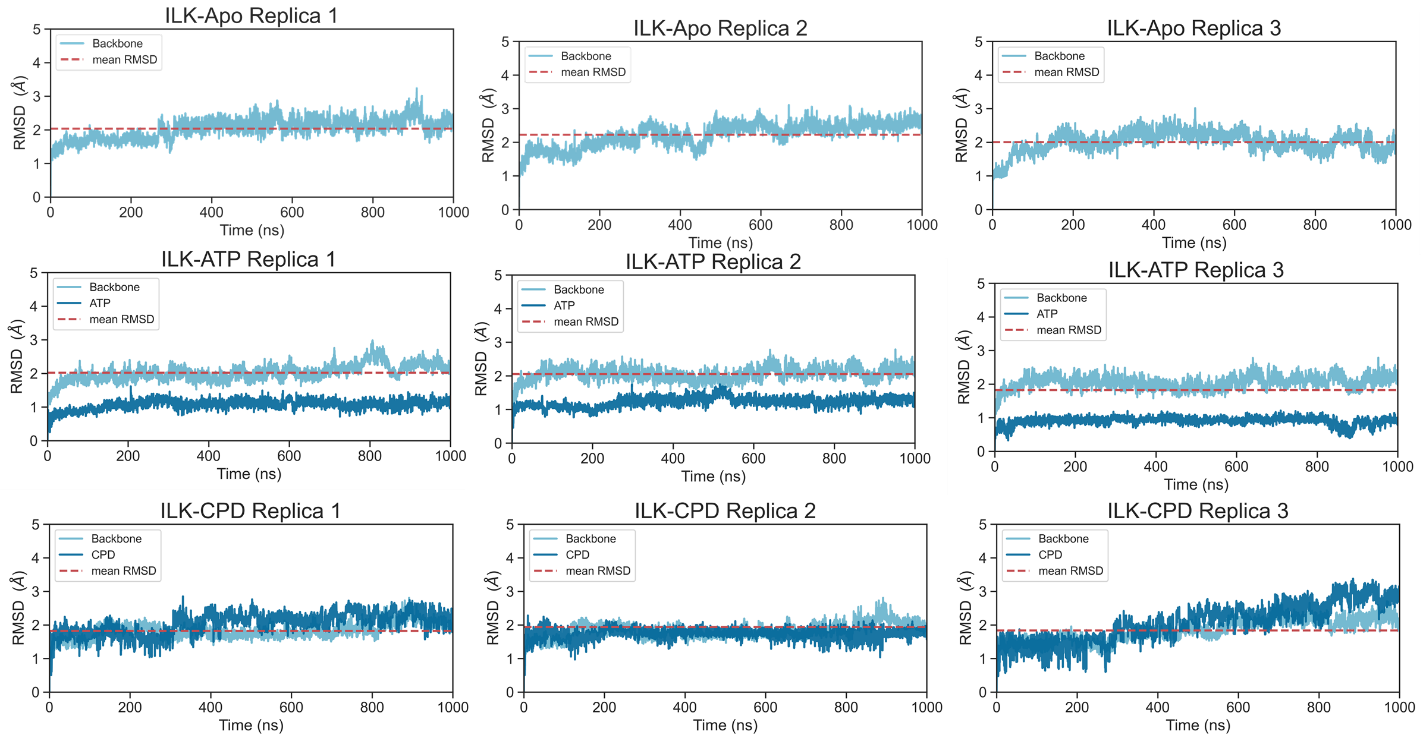


**Fig. S 5** Plots with the root mean square deviations (RMSD, Å) of protein (backbone) and ligands (heavy atoms) during MD simulations. The red dashed line represents the mean RMSD value of the protein.


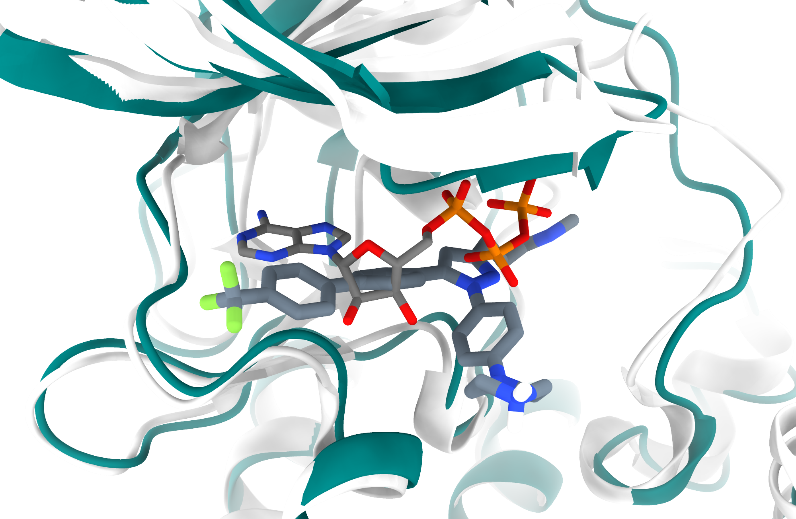


**Fig. S 6** Superimposition of ILK crystal structure bound to ATP (PDB ID: 3KMW) onto a representative snapshot of the most populated cluster obtained from molecular dynamics of Cpd22 in complex with ILK.


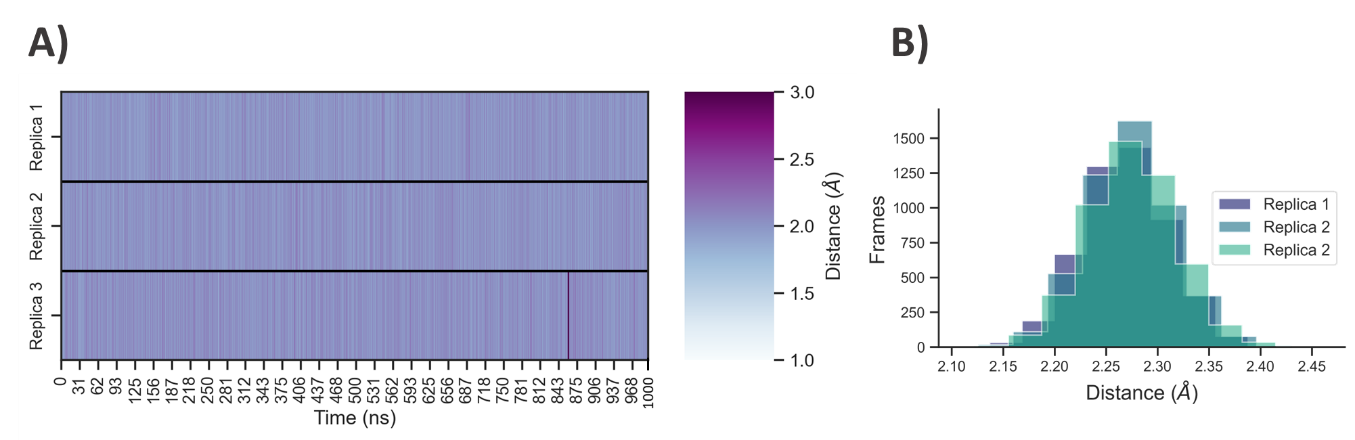


**Fig. S 7** A) Heatmap plot for distance between Oxygen atom from Cpd22 carbonyl group and the Mg^2+^ cation present in the pseudo-active site of ILK during MD simulations. B) Distance values distribution for three replicas.

**Table S 1.** Protein radius of gyration mean values across trajectories (Å).

|  | **Replica1** | **Replica1** | **Replica 2** | **Media** | **SD** |
| --- | --- | --- | --- | --- | --- |
|  |  |  |  |  |  |
| **Apo** | 9.02 | 18.97 | 19.01 | 19.00 | 0.08 |
| **ATP** | 19.15 | 19.15 | 18.96 | 19.09 | 0.10 |
| **Cpd22** | 19.20 | 19.11 | 19.09 | 19.13 | 0.09 |


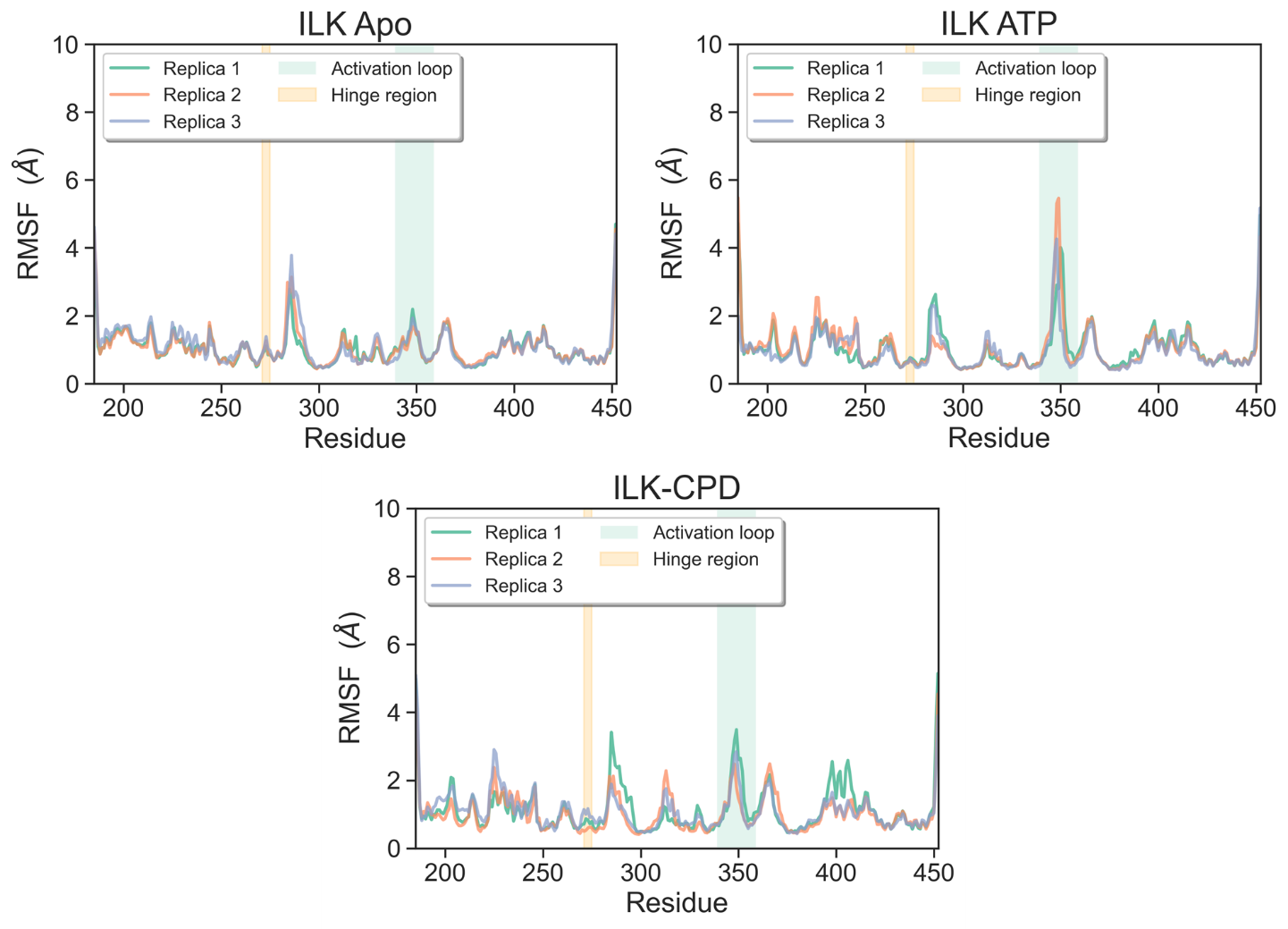


**Fig. S 8** Comparisons of apo-ILK, ILK-ATP and ILK-Cpd22 protein Cα carbons RMSF across replicas.

**Table S 2** Predicted binding free energy for Cpd22 and ATP binding to ILK by MM-ISMSA algorithm (kcal/mol).

| **Replica** | **Total** | **VDW** | **QQ** | **DesolvLigand** | **DesolvReceptor** | **Apolar** | |
| --- | --- | --- | --- | --- | --- | --- | --- |
| **ATP** | | | | | | |  |
| **1** | -36.11 | -11.48 | -35.90 | 12.74 | 2.32 | -3.79 | |
| **2** | -51.29 | -16.60 | -31.50 | 11.65 | -10.31 | -4.54 | |
| **3** | -64.08 | -12.81 | -38.17 | 11.56 | -20.49 | -4.17 | |
| **Cpd22** | | | | | | |  |
| **1** | -54.12 | -51.15 | -14.45 | -1.50 | 17.47 | -4.48 | |
| **2** | -41.82 | -42.93 | -13.61 | 2.13 | 16.54 | -3.95 | |
| **3** | -50.85 | -45.18 | -15.35 | -3.74 | 17.63 | -4.21 | |


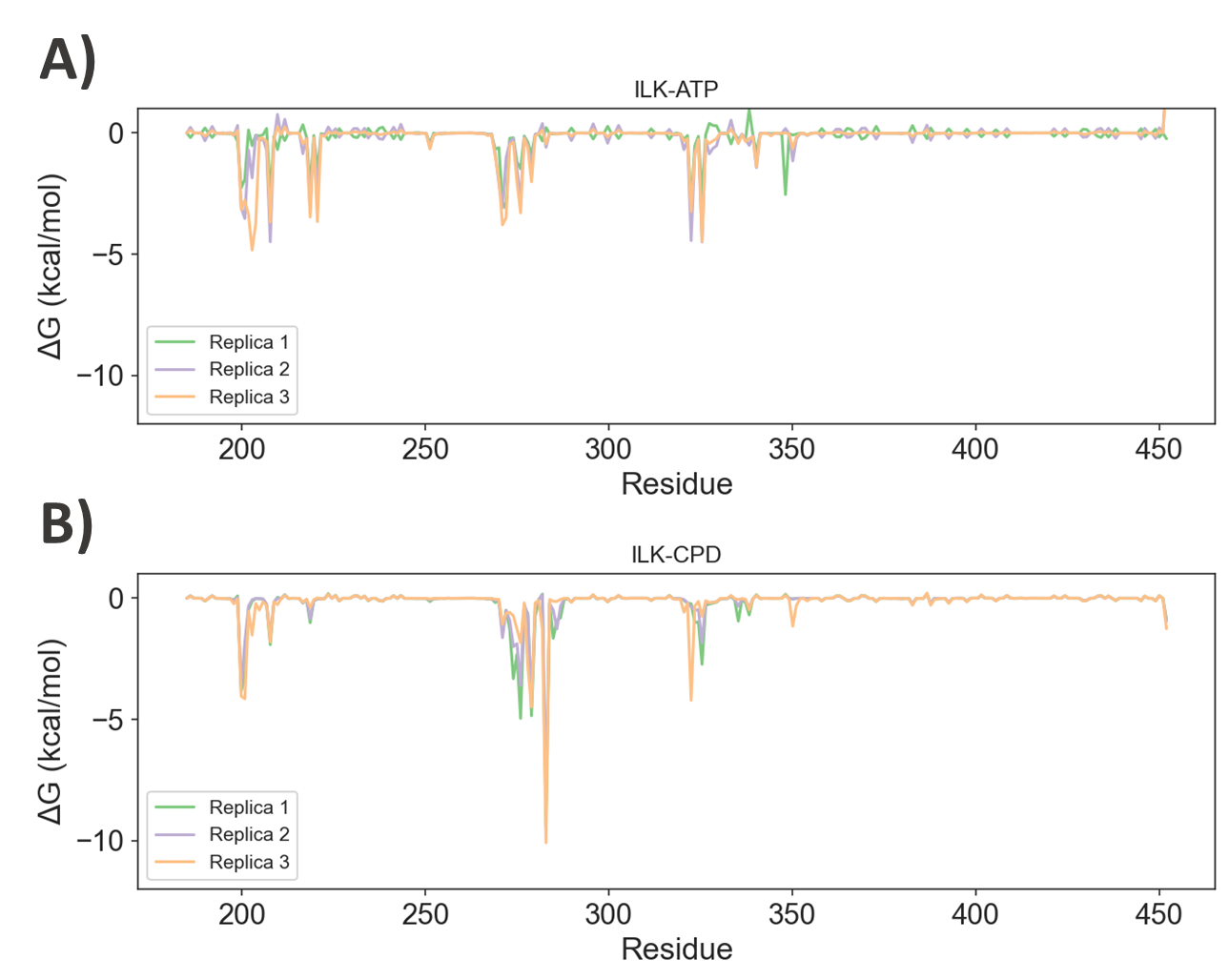


**Fig. S 8** Residues contributing to the binding of ATP (A) or Cpd22 (B) with the value of the binding energy to ILK.

**SMILES codes**:

C1CNCCN1c(cc2)ccc2-n(nc3CCC(=O)NC)c(c3)-c4ccc(cc4)-c5ccc(C(F)(F)F)cc5,Cpd22

C1C[NH2+]CCN1c(cc2)ccc2-n(nc3CCC(=O)NC)c(c3)-c4ccc(cc4)-c5ccc(C(F)(F)F)cc5,Cpd22-H
